# Celebrating 75 years of the *Journal of Experimental Botany* (1950–2025)

**DOI:** 10.1093/jxb/eraf295

**Published:** 2025-09-03

**Authors:** John E Lunn

**Affiliations:** Max Planck Institute of Molecular Plant Physiology, 14476, Potsdam-Golm, Germany

**Since its foundation in 1950, the *Journal of Experimental Botany* (*JXB*) has been publishing articles at the forefront of plant science for 75 years. To celebrate this significant milestone in the journal’s history, we commissioned a special series of our flagship Darwin Reviews from leading plant scientists to take a snapshot of plant science today, across a broad range of topics. The reviews range from the molecular and cellular levels of metabolism and signalling (Kopriva *et al*., 2024; Smirnoff and Wheeler, 2024; Witte and Herde, 2024; Fuertes-Rabanal *et al*., 2025; John *et al.*, 2025) to the organ and whole-plant levels of growth and development (Mody *et al.*, 2025; Pandey *et al.*, 2025). With world agriculture facing the dual challenges of feeding a growing population and global climate change, there is a particular focus on crop species and strategies for improving resilience to abiotic and biotic stresses (Jaryal *et al.*, 2025; Munzert and Engelsdorf, 2025; Prado *et al*., 2025) and optimising growth and development (Robil *et al.*, 2025), as well as the potential for diversification into new crop species (Phillips *et al.*, 2025).**

*JXB* was founded in 1950 by the Society for Experimental Biology (SEB), a learned society for animal, cell and plant biologists, to meet the needs of a growing community of experimental plant scientists to publish their work. Although *JXB* had British roots, it soon became recognised as a leading international journal for the publication of high-quality plant research. This is reflected in the geographic diversity of our authorship and editorial board, with members from nearly 30 countries representing all four corners of the world. *JXB* continues to be owned and run by the SEB and income from the journal is used for the benefit of the scientific community, providing financial support for SEB conferences and workshops as well as the society’s charitable activities, such as grants and travel awards for early career researchers.

With generous financial and logistical support from the SEB, *JXB* is organising a symposium in Edinburgh (17–19 September, 2025) to mark the journal’s 75^th^ anniversary (https://www.sebiology.org/events/journal-of-experimental-botany-symposium.html). The meeting will cover a broad range of topics, with the aim of bringing together plant researchers from different disciplines, and will provide opportunities for young researchers to present their work alongside world-leading plant scientists. There will also be workshops on scientific ethics and publishing, along with social events for making scientific and personal connections. In the final session, we shall be looking to the future of plant science in the next 25 years, with the hope that *JXB* will continue to play a leading role in the dissemination of cutting-edge research and support for the plant research community as the journal approaches its 100^th^ birthday.
